# Three-Year Follow-Up of High-Dose Ubiquinol Supplementation in a Case of Familial Multiple System Atrophy with Compound Heterozygous *COQ2* Mutations

**DOI:** 10.1007/s12311-017-0846-9

**Published:** 2017-02-01

**Authors:** Jun Mitsui, Ken Koguchi, Toshimitsu Momose, Miwako Takahashi, Takashi Matsukawa, Tsutomu Yasuda, Shin-ichi Tokushige, Hiroyuki Ishiura, Jun Goto, Shigeaki Nakazaki, Tomoyoshi Kondo, Hidefumi Ito, Yorihiro Yamamoto, Shoji Tsuji

**Affiliations:** 1 Department of Neurology, Graduate School of Medicine, University of Tokyo, 7-3-1 Hongo, Bunkyo, Tokyo, 113-8655 Japan; 2 Department of Neurology, Shirahama Hamayu Hospital, Wakayama, Japan; 3 Department of Radiology, Graduate School of Medicine, University of Tokyo, Tokyo, Japan; 4 Department of Neurology, Rehabilitation Hananoie Hospital, Tochigi, Japan; 5 Department of Neurology, Wakayama Medical University, Wakayama, Japan; 6 School of Bioscience and Biotechnology, Tokyo University of Technology, Tokyo, Japan

**Keywords:** Multiple system atrophy, *COQ2*, Coenzyme Q_10_, Ubiquinol

## Abstract

We report a 3-year follow-up of high-dose ubiquinol supplementation in a case of familial multiple system atrophy (MSA) with compound heterozygous nonsense (R387X) and missense (V393A) mutations in *COQ2*. A high-dose ubiquinol supplementation substantially increased total coenzyme Q_10_ levels in cerebrospinal fluid as well as in plasma. The patient was at the advanced stage of MSA, and the various scores of clinical rating scales remained stable without changes during the 3 years. The cerebral metabolic ratio of oxygen measured by ^15^O_2_ PET, however, increased by approximately 30% after administration of ubiquinol, suggesting that ubiquinol can improve mitochondrial oxidative metabolism in the brain. It also suggests the therapeutic potential of ubiquinol for patients with MSA with *COQ2* mutations. Further clinical trials of administration of high-dose ubiquinol to MSA patients are warranted.

## Introduction

Multiple system atrophy (MSA) is a progressive neurodegenerative disease clinically characterized by autonomic failure in addition to various combinations of parkinsonism, cerebellar ataxia, and pyramidal dysfunction [1]. Whole-genome sequence analysis in combination with linkage analysis has revealed homozygous or compound heterozygous mutations in *COQ2* in two of the six multiplex Japanese families with MSA [2]. *COQ2* encodes an enzyme in the biosynthetic pathway of coenzyme Q_10_ (CoQ_10_) [3]. Indeed, the total CoQ_10_ levels in frozen brain tissues and lymphoblastoid cell lines from patients with MSA carrying homozygous (M128V-V393A/M128V-V393A) and compound heterozygous mutations (R387X/V393A) were substantially lower than those from control subjects [2]. These observations suggest the efficacy of CoQ_10_ supplementation for patients with MSA, in particular, for those with *COQ2* mutations.

CoQ_10_ is a lipophilic molecule that functions as an essential carrier for electron transport in the mitochondrial respiratory chain and as an endogenous antioxidant [4]. Patients with primary CoQ_10_ deficiency, a severer phenotype than MSA, caused by genetic defects in genes involved in the CoQ_10_ biosynthetic pathway, have been reported to respond well to CoQ_10_ supplementation [5–11]. Herein, we present the detailed clinical features of a patient with familial MSA carrying compound heterozygous *COQ2* mutations (R387X/V393A) [2] and report the outcome of a high-dose ubiquinol (reduced form of CoQ_10_) supplementation to this patient.

## Materials and Methods

### Patient

The patient, a 60-year-old male, is an affected member of a previously described Japanese multiplex family with MSA (II-4 in Family 12) [2]. He carried compound heterozygous nonsense (R387X, c.1159C>T) and missense (V393A, c.1178T>C) mutations in *COQ2* (NM_015697.7) [2, 12].

The patient gradually noticed slurring of speech, unsteadiness of gait, increased urinary frequency, and erectile dysfunction at the age of 44. Examination at the age of 45 revealed jerky pursuit of eye movements, gaze-evoked nystagmus, and scanning speech. Lower limb movements were uncoordinated when performing the heel-knee-shin test. His gait was ataxic, and he was unable to perform tandem gait. He had urinary urgency, frequent urination, and erectile dysfunction. He also had frequent orthostatic symptoms attributable to hypotension. His cognition and visual acuity were normal. Brain magnetic resonance imaging (MRI) showed the hot cross bun sign in the pontine base and mild atrophy of the pons and cerebellum. He was then diagnosed as having “familial MSA” because his elder sister had a similar presentation with dysarthria and unsteady gait with the onset at the age of 50. She died at the age of 61. He started intermittent catheterization at the age of 47 and had frequent orthostatic symptoms once a week at the age of 48. He became wheelchair-bound at the age of 50 and subsequently became bed-ridden owing to his severe orthostatic symptoms. At the age of 56, his speech was unintelligible most of the time due to the severe ataxic dysarthria. At the age of 58, endoscopic gastrostomy and laryngotracheal separation were performed owing to his recurrent aspiration pneumonia. At the age of 59, his renal function gradually worsened (serum creatinine level, 1.0–1.5 mg/dl).

### Study Design

An open-label dose escalation trial for this patient with familial MSA with compound heterozygous *COQ2* mutations (R387X/V393A) was designed to evaluate the safety and tolerability of high-dose ubiquinol, to assess pharmacokinetics, and to obtain the clinical data including the scores of clinical rating scales [Barthel Index [13], Scale for the Assessment and Rating of Ataxia (SARA) [14], International Cooperative Ataxia Rating Scale (ICARS) [15], and Unified Multiple System Atrophy Rating Scale (UMSARS) [16]]. Positron emission tomography (PET) was carried out to measure the cerebral blood flow (CBF) and the cerebral metabolic rate of oxygen (CMRO_2_). The protocol of this study (UMIN000010712) was reviewed and approved by the institutional review board of the participating institutions. Informed consent was obtained from the patient and his legal representative prior to the initiation of this study, in accordance with the Declaration of Helsinki. Ubiquinol in the powder form (Kaneka QH ubiquinol) was provided by Kaneka Corporation (Tokyo, Japan) and was administered via a gastrostomy tube.

After baseline assessment, supplementation was started at 600 mg of ubiquinol/day (given once a day), with the dosage increased to 840 mg/day at week 2 and to 1200 mg/day at week 6. The 1200-mg/day dosage was maintained until week 8. When no adverse events were observed during this period, the patient resumed taking 1200 mg of ubiquinol/day after an interval of 8 weeks and remained taking ubiquinol at this same dose for over 3 years to date.

### Biochemical Analysis

Peripheral blood was collected into heparinized tubes to obtain plasma samples. BD Vacutainer CPT Cell Preparation Tubes (BD, Franklin Lakes, NJ) with sodium heparin were used for separation of peripheral blood mononuclear cells (PBMCs). The total CoQ_10_ levels (sum of ubiquinol and ubiquinone levels) in plasma, PBMCs, and cerebrospinal fluid (CSF) were measured by high-performance liquid chromatography (HPLC) with electrochemical detection (ECD) [17] or with tandem-mass spectrometry (LC-MS/MS). For measurement of the total CoQ_10_ level in CSF by HPLC with ECD, 400 μl of CSF was mixed with 1600 μl of isopropanol. An aliquot of 1500 μl was evaporated to dryness and resolved in 120 μl of isopropanol and 30 μl of distilled water. An aliquot of 80 μl of the supernatant was directly injected onto the HPLC equipped with the separation column (SHISEIDO CQ-S: 150 mm × 2.0 mm I.D.) with the mobile phase of 50 mM sodium perchlorate in methanol/distilled water (95/5, *v*/*v*). Measurement of the total CoQ_10_ level in additional CSF samples obtained after 12, 24, and 36 months of ubiquinol supplementation was conducted as follows. Twenty microliters of CSF was diluted to 5 ml with phosphate-buffered saline and mixed with 8 ml of diethyl ether and 10 μl of internal standard solution (200 ng/ml of CoQ_8_ in isopropanol). After the solution was vigorously mixed, the supernatant separated by centrifugation was evaporated to dryness and resolved in 20 μl of isopropanol. Quantitation of CoQ_10_ was accomplished by LC-MS/MS using Nexera X2 and LCMS-8060 (Shimadzu, Japan).

### Measurement of the CBF and the CMRO_2_ by PET

PET studies were carried out with ^15^O-labeled tracers (^15^O_2_, C^15^O_2_, and C^15^O). The C^15^O_2_ and ^15^O_2_ steady-state methods were used to measure the CBF and CMRO_2_, respectively [18, 19]. PET scanning was performed during C^15^O_2_ (370 MBq/min) or ^15^O_2_ (740 MBq/min) gas inhalation after equilibrium had been reached. C^15^O PET (1110 MBq/min) was performed to measure cerebral blood volume (CBV) that was used for correction of CMRO_2_ [19–21]. PET imaging was performed using a PET scanner (Headtome/SET2400 W, Shimadzu).

For quantitative analysis, brain PET images before and after ubiquinol supplementation were intrasubjectively coregistered and morphologically normalized to the brain PET template using statistical parametric mapping 8 and MATLAB version R2014a (MathWorks Inc., Natick, MA, USA). The regions of interest (ROIs) were manually placed on the cortical ribbon of the upper frontal, lower frontal, Rolandic, lateral and medial parietal, temporal, and occipital areas and on the striatum, thalamus, cerebellar hemisphere, and cerebellar vermis on the morphologically normalized CBF PET images obtained before supplementation. These ROIs were automatically applied to other PET images, and each ROI value was then computationally calculated.

## Results

### Ubiquinol Supplementation and Clinical Outcome

The analysis of the total CoQ_10_ levels in plasma and PBMCs revealed a significant increase after 2 weeks of ubiquinol supplementation at 600 mg/day (Table 1). The total CoQ_10_ levels in plasma and PBMCs remained similar for another 4 weeks at 840 mg/day, and a subsequent 2-week administration of ubiquinol at 1200 mg/day led to substantial increases in the total CoQ_10_ levels in the plasma and the PBMCs. The CoQ_10_ level in CSF increased from 0.22 to 3.79 ng/ml after 2 weeks of 840 mg/day, and a similar level of 3.64 ng/ml was observed after 2 weeks of 1200 mg/day. Eight weeks after the last supplementation of ubiquinol, the total CoQ_10_ levels in plasma, PBMCs, and CSF returned to baseline levels.

**Table 1:** Biochemical analyses

	Total CoQ_10_ (μg/ml) in plasma	Total CoQ_10_/free cholesterol in PBMCs (nM/mM)	Total CoQ_10_ in CSF (μg/ml)
Reference (mean, standard deviation, number of controls)	0.72, 0.42, *n* = 39 (healthy controls) [31]	Not available	0.35 × 10^−3^, 0.20 × 10^−3^, *n* = 23 (disease controls)
Before supplementation	0.33	281	0.22 × 10^−3^
After 2 weeks of 600 mg/day	5.04	1493	Not tested
After 2 weeks of 840 mg/day	4.02	1344	3.79 × 10^−3^
After 4 weeks of 840 mg/day	4.43	1636	Not tested
After 2 weeks of 1200 mg/day	7.86	2047	3.64 × 10^−3^
After 8 weeks of discontinuation	0.48	467	0.25 × 10^−3^
After 6 months of 1200 mg/day	4.15	1894	Not tested
After 12 months of 1200 mg/day	7.62	1891	7.36 × 10^−3a^
After 24 months of 1200 mg/day	4.92	Not tested	9.14 × 10^−3a^
After 36 months of 1200 mg/day	4.78	Not tested	14.06 × 10^−3a^

Total CoQ_10_, ubiquinol + ubiquinone

^a^Measured by LC-MS/MS

Since we did not observe any adverse events at 1200 mg/day dosage and ubiquinol supplementation at 1200 mg/day led to higher total CoQ_10_ levels in the plasma and the PBMCs compared with those observed with 840 mg/day, we decided to maintain the 1200-mg/day dosage. After supplementation of 1200 mg/day was resumed, the CoQ_10_ levels in plasma and PBMCs returned to levels similar to those observed after the initial 2 weeks of 1200 mg/day and maintained at similar levels throughout the following period. The CoQ_10_ levels in CSF were 7.36, 9.14, and 14.06 ng/ml after 12, 24, and 36 months of 1200 mg/day, respectively. Thus, the total CoQ_10_ levels in CSF gradually increased after resuming ubiquinol supplementation at 1200 mg/day for the following period (Table 1).

**Fig. 1:**
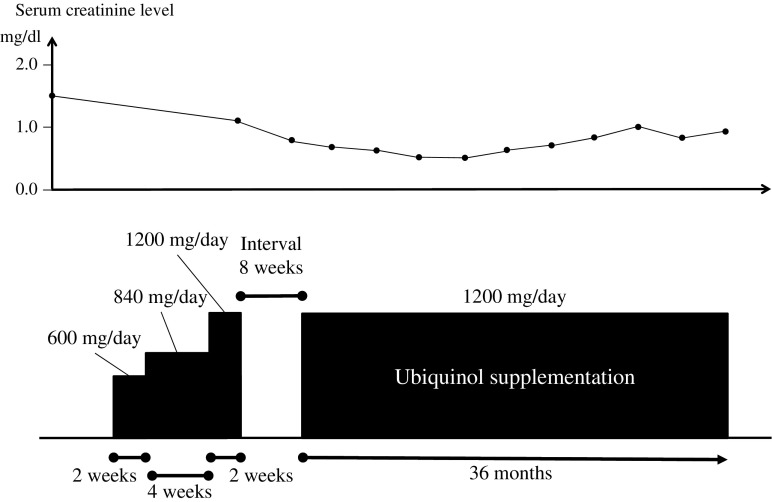
Summary of clinical course with ubiquinol supplementation. After the baseline assessment, the patient was started on ubiquinol at 600 mg/day (given once a day), with the dose increased to 840 mg/day at week 2 and to 1200 mg/day at week 6. The 1200-mg/day dose was maintained until week 8. After an interval of 8 weeks, the patient resumed taking ubiquinol at 1200 mg/day. To date, he remains taking ubiquinol at this same dose for over 3 years. *BW* body weight, *sCre* serum creatinine level

**Fig. 2:**
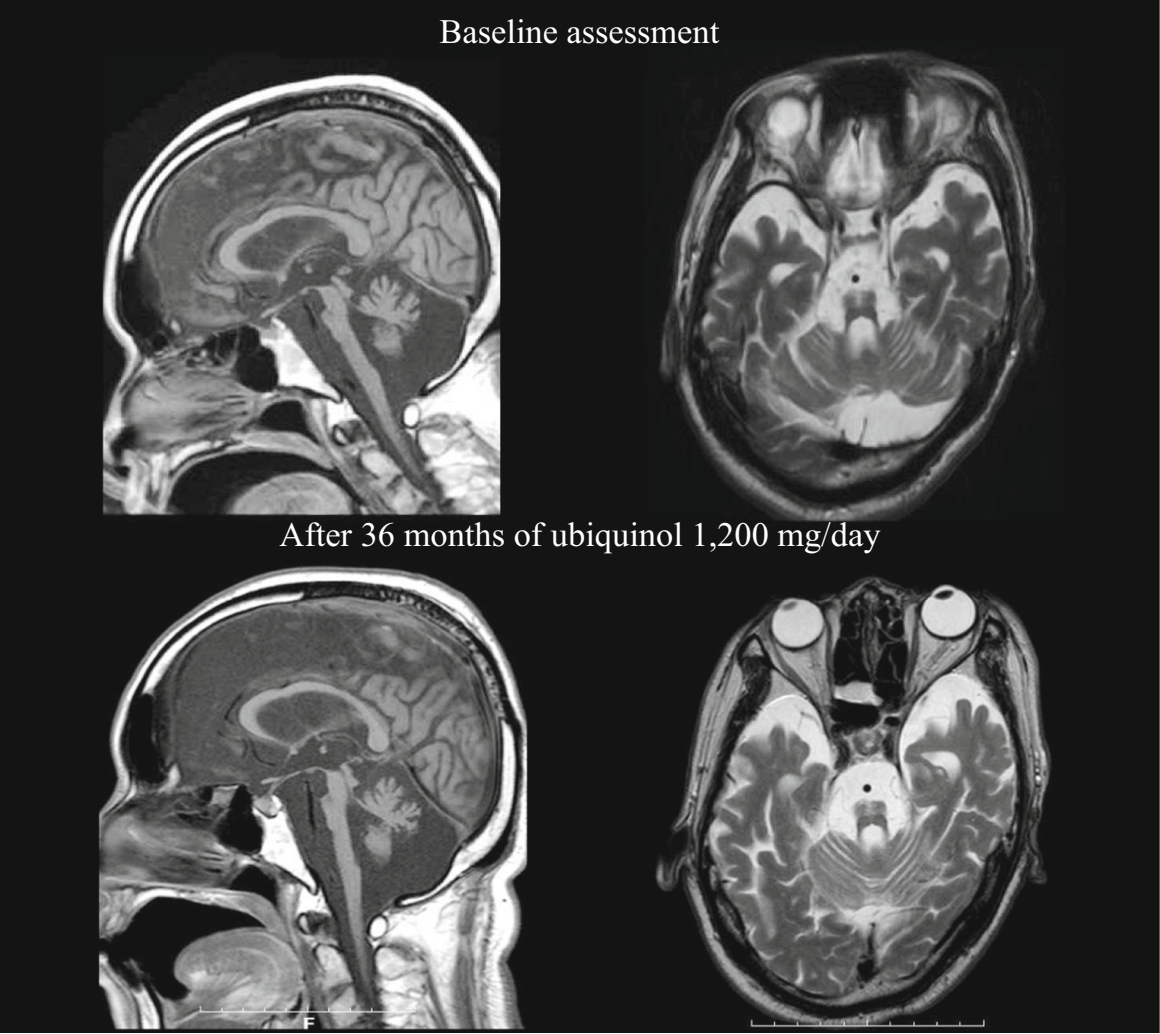
Brain MR images of patient. Upper images were obtained at 60 years of age (before supplementation of ubiquinol), and lower images were obtained at 63 years of age (after 36 months of ubiquinol 1200 mg/day). T1-weighted sagittal images (*left*) and T2-weightd axial images at the middle regions of the pontine base (*right*). They show gross atrophy of the infratentorial structures especially in the pons, middle cerebellar peduncle, and cerebellum

The patient continued to take 1200 mg of ubiquinol/day for over 3 years (Fig. 1). During the entire course, we did not observe any adverse events that were considered to be associated with the ubiquinol supplementation throughout the entire study period. After 36 months of supplementation, evaluation of scores of clinical rating scales (Barthel index, SARA, ICARS, and UMSARS) showed no remarkable changes (Table 2). The brain MRI findings also remained unchanged for the 3 years (Fig. 2). It was notable that his serum creatinine level gradually declined over 36 months (from 1.45 to 0.95 mg/dl) (Fig. 1). His body weight decreased in the first 16 weeks (48.5 to 41.0 kg), but gradually increased over 36 months (41.0 to 46.0 kg) after increasing his daily calorie intake (from 900 to 1500 kcal/day).

**Table 2:** Scores of clinical rating scales

	Barthel index	SARA	ICARS (dynamic)	ICARS (static)	UMSARS Part I	UMSARS Part II	UMSARS Part IV
Before supplementation	0	40	50	34	47	49	5
After a 2-week supplementation at 600 mg/day	0	40	50	34	47	47	5
After a 4-week supplementation at 840 mg/day	0	40	50	34	47	51	5
After a 2-week supplementation at 1200 mg/day	0	39	50	34	47	49	5
After a 6-month supplementation at 1200 mg/day	0	39	51	34	47	50	5
After a 12-month supplementation at 1200 mg/day	0	39	51	34	47	51	5
After an 18-month supplementation at 1200 mg/day	0	40	52	34	47	51	5
After a 24-month supplementation at 1200 mg/day	0	40	51	34	47	50	5
After a 30-month supplementation at 1200 mg/day	0	40	50	34	47	49	5
After a 36-month supplementation at 1200 mg/day	0	40	50	34	47	49	5

### Measurement of the CBF and the CMRO_2_ by PET

**Fig. 3:**
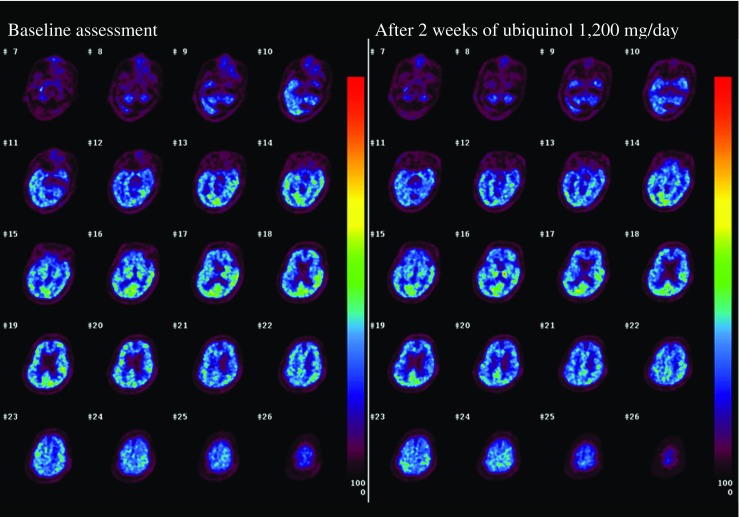
CBF images before and after ubiquinol supplementation at 1200 mg/day

**Fig. 4:**
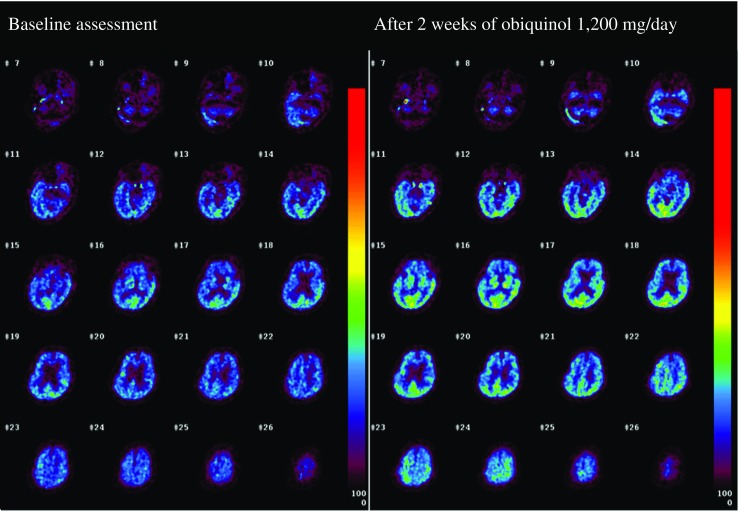
CMRO_2_ images before and after ubiquinol supplementation at 1200 mg/day

Figures 3 and 4 show CBF and CMRO_2_ images, respectively, and Table 3 summarizes the quantitative measurements before and after 2 weeks of ubiquinol supplementation at 1200 mg/day. At the baseline, CBF decreased and CMRO_2_ markedly decreased in the entire brain. For example, the CBF and CMRO_2_ in the Rolandic area were 30.6 ml/100 ml/min [reference level [19], 44.6, 5.6 (mean, standard deviation)] and 1.81 ml/100 ml/min [reference level [19], 3.3, 0.5 (mean, standard deviation)], respectively. After 2 weeks of ubiquinol supplementation at 1200 mg/day, CBF remained unchanged as compared with the baseline level (Fig. 3 and Table 3). In contrast, the CMRO_2_ markedly increased by approximately 30% in the entire brain (Fig. 4 and Table 3), although it did not reach normal levels. For example, the CMRO_2_ in the Rolandic area increased from 1.81 to 2.15 ml/100 ml/min [reference level, 3.3, 0.5 (mean, standard deviation) [19]].

**Table 3:** CBF and CMRO_2_ in major brain areas before and after ubiquinol supplementation

	CBF (ml/100 ml/min)		CMRO_2_ (ml/100 ml/min)	
Region	Before	After	Before	After
Upper frontal	28.8	27.7	1.78	2.17
Lower frontal	29.7	29.8	1.72	2.23
Rolandic	30.6	29.7	1.81	2.15
Lateral parietal	30.8	29.9	2.03	2.52
Medial parietal	35.3	42.1	2.05	3.12
Temporal	31.5	30.3	2.11	2.65
Occipital	38.2	37.5	2.63	3.17
Striatum	30.4	29.7	1.92	2.56
Thalamus	35.3	44.5	2.08	3.20
Cerebellar hemisphere	24.3	24.3	1.44	1.89
Cerebellar vermis	23.7	22.4	1.33	1.58

## Discussion

In the present single case study of a patient with familial MSA carrying compound heterozygous mutations in *COQ2*, administration of high-dose ubiquinol led to a substantial increase in the total CoQ_10_ levels not only in the plasma and PBMC but also in the CSF. Although previous reports have failed to show the increase in total CSF CoQ_10_ level by ubiquinone or ubiquinol supplementation, which was caused possibly due to the insufficient dose (300 mg/day) [25], this is the first study showing that ubiquinol supplementation at 840 and 1200 mg/day clearly elevated the total CSF CoQ_10_ level. CoQ_10_ has been reported to be poorly absorbed, and its bioavailability varies among formulations [26]. Previous dose escalation studies (up to 3000 mg/day) using chewable tablets of ubiquinone in patients with Parkinson disease, amyotrophic lateral sclerosis, and Huntington disease concordantly showed that the total plasma CoQ_10_ levels reached the plateau levels of approximately 7.0–7.5 μg/ml after multiple doses of 2400 mg/day [22–24]. When assessing the bioavailability of ubiquinol in this study, the trough concentrations of total CoQ_10_ in plasma were 5.04 μg/ml for 600 mg/day, 4.02 μg/ml for 840 mg/day, and 7.86 μg/ml for 1200 mg/day 2 weeks after the daily intake of ubiquinol. Furthermore, another previous study using ubiquinol showed that mean total plasma CoQ_10_ levels were 2.61 μg/ml for 90 mg/day, 3.66 μg/ml for 150 mg/day, and 6.53 μg/ml for 300 mg/day 2 weeks after a daily intake of ubiquinol [27]. These observations indicate that ubiquinol is better absorbed in the gastrointestinal tract than ubiquinone, and we conclude that the ubiquinol dose of 1200 mg/day is sufficient for achieving a plateau of total CoQ_10_ level in plasma.

Remarkably, CMRO_2_ increased without an increase in CBF after administration of 1200 mg of ubiquinol, which suggests that ubiquinol improved cerebral mitochondrial oxidative metabolism. Despite the increase in the CMRO_2_, however, we did not detect any obvious neurological improvements as determined by the rating scales, presumably owing to the advanced stage of neurodegeneration. Notably, his serum creatinine level gradually declined during the ubiquinol supplementation over 36 months (from 1.45 to 0.95 mg/dl). Because renal involvement has been frequently observed in patients with primary CoQ_10_ deficiency caused by genetic defects in CoQ_10_ biosynthesis [28–30], the renal dysfunction in the patient was likely caused by CoQ_10_ deficiency and was ameliorated by ubiquinol supplementation. He also showed weight loss in the first 16 weeks of supplementation (48.5 to 41.0 kg). We extensively investigated the cause of his weight loss. However, we did not find chronic infectious diseases, malignancies, extremity edema, pleural effusion, or ascites in this patient during the entire study period. He gradually regained his body weight over 36 months (41.0 to 46.0 kg) after increasing his daily calorie intake. Despite the body weigh changes, his general health condition remained stable.

## Conclusions

The current study suggests that high-dose ubiquinol supplementation (up to 1200 mg/day) is tolerable and improves cerebral mitochondrial oxidative metabolism, which may alter the natural history of MSA progression especially when applied in the early phase of MSA in patients with genetic defects in the CoQ_10_ biosynthetic pathway. Further clinical trials including administration of ubiquinol to MSA patients carrying heterozygous *COQ2* mutations as well as to patients without mutations in *COQ2* are warranted. Prospective randomized controlled trials will be undertaken to further extend these initial promising observations.

## References

[1] Gilman S, Wenning GK, Low PA, Brooks DJ, Mathias CJ, Trojanowski JQ (2008). Second consensus statement on the diagnosis of multiple system atrophy. Neurology.

[2] The Multiple-System Atrophy Research Collaboration (2013). Mutations in COQ2 in familial and sporadic multiple-system atrophy. N Engl J Med.

[3] Ashby MN, Kutsunai SY, Ackerman S, Tzagoloff A, Edwards PA (1992). COQ2 is a candidate for the structural gene encoding para-hydroxybenzoate:polyprenyltransferase. J Biol Chem.

[4] Littarru GP, Tiano L. Bioenergetic and antioxidant properties of coenzyme Q10: recent developments. Mol Biotechnol. 2007;37:31–7. 10.1007/s12033-007-0052-y 17914161

[5] Lagier-Tourenne C, Tazir M, Lopez LC, Quinzii CM, Assoum M, Drouot N (2008). ADCK3, an ancestral kinase, is mutated in a form of recessive ataxia associated with coenzyme Q10 deficiency. Am J Hum Genet.

[6] Mollet J, Delahodde A, Serre V, Chretien D, Schlemmer D, Lombes A (2008). CABC1 gene mutations cause ubiquinone deficiency with cerebellar ataxia and seizures. Am J Hum Genet.

[7] Pineda M, Montero R, Aracil A, O’Callaghan MM, Mas A, Espinos C (2010). Coenzyme Q(10)-responsive ataxia: 2-year-treatment follow-up. Mov Disord.

[8] Liu YT, Hersheson J, Plagnol V, Fawcett K, Duberley KE, Preza E (2014). Autosomal-recessive cerebellar ataxia caused by a novel ADCK3 mutation that elongates the protein: clinical, genetic and biochemical characterisation. J Neurol Neurosurg Psychiatry.

[9] Mignot C, Apartis E, Durr A, Marques Lourenço C, Charles P, Devos D (2013). Phenotypic variability in ARCA2 and identification of a core ataxic phenotype with slow progression. Orphanet J Rare Dis.

[10] Rotig A, Appelkvist EL, Geromel V, Chretien D, Kadhom N, Edery P (2000). Quinone-responsive multiple respiratory-chain dysfunction due to widespread coenzyme Q10 deficiency. Lancet.

[11] Van Maldergem L, Trijbels F, DiMauro S, Sindelar PJ, Musumeci O, Janssen A (2002). Coenzyme Q-responsive Leigh’s encephalopathy in two sisters. Ann Neurol.

[12] Hara K, Momose Y, Tokiguchi S, Shimohata M, Terajima K, Onodera O (2007). Multiplex families with multiple system atrophy. Arch Neurol.

[13] Mahoney FI, Barthel DW (1965). Functional evaluation: the Barthel index. Md State Med J.

[14] Schmitz-Hübsch T, du Montcel ST, Baliko L, Berciano J, Boesch S, Depondt C (2006). Scale for the assessment and rating of ataxia: development of a new clinical scale. Neurology.

[15] Trouillas P, Takayanagi T, Hallett M, Currier RD, Subramony SH, Wessel K (1997). International Cooperative Ataxia Rating Scale for pharmacological assessment of the cerebellar syndrome. The Ataxia Neuropharmacology Committee of the World Federation of Neurology. J Neurol Sci.

[16] Wenning GK, Tison F, Seppi K, Sampaio C, Diem A, Yekhlef F (2004). Development and validation of the Unified Multiple System Atrophy Rating Scale (UMSARS). Mov Disord.

[17] Yamashita S, Yamamoto Y (1997). Simultaneous detection of ubiquinol and ubiquinone in human plasma as a marker of oxidative stress. Anal Biochem.

[18] Frackowiak RS, Lenzi GL, Jones T, Heather JD (1980). Quantitative measurement of regional cerebral blood flow and oxygen metabolism in man using 15O and positron emission tomography: theory, procedure, and normal values. J Comput Assist Tomogr.

[19] Ito H, Kanno I, Kato C, Sasaki T, Ishii K, Ouchi Y (2004). Database of normal human cerebral blood flow, cerebral blood volume, cerebral oxygen extraction fraction and cerebral metabolic rate of oxygen measured by positron emission tomography with 15O-labelled carbon dioxide or water, carbon monoxide and oxygen. Eur J Nucl Med Mol Imaging.

[20] Martin WR, Powers WJ, Raichle ME (1987). Cerebral blood volume measured with inhaled C15O and positron emission tomography. J Cereb Blood Flow Metab.

[21] Lammertsma AA, Jones T (1983). Correction for the presence of intravascular oxygen-15 in the steady-state technique for measuring regional oxygen extraction ratio in the brain: 1. Description of the method. J Cereb Blood Flow Metab.

[22] Shults CW, Flint Beal M, Song D, Fontaine D (2004). Pilot trial of high dosages of coenzyme Q10 in patients with Parkinson’s disease. Exp Neurol.

[23] Hyson HC, Kieburtz K, Shoulson I, McDermott M, Ravina B, de Blieck EA (2010). Safety and tolerability of high-dosage coenzyme Q10 in Huntington’s disease and healthy subjects. Mov Disord.

[24] Ferrante KL, Shefner J, Zhang H, Betensky R, O’Brien M, Yu H (2005). Tolerance of high-dose (3,000 mg/day) coenzyme Q10 in ALS. Neurology.

[25] Lönnrot K, Metsä-Ketelä T, Molnár G, Ahonen JP, Latvala M, Peltola J (1996). The effect of ascorbate and ubiquinone supplementation on plasma and CSF total antioxidant capacity. Free Radic Biol Med.

[26] Bhagavan HN, Chopra RK (2007). Plasma coenzyme Q10 response to oral ingestion of coenzyme Q10 formulations. Mitochondrion.

[27] Hosoe K, Kitano M, Kishida H, Kubo H, Fujii K, Kitahara M (2007). Study on safety and bioavailability of ubiquinol (Kaneka QH) after single and 4-week multiple oral administration to healthy volunteers. Regul Toxicol Pharmacol.

[28] Lopez LC, Schuelke M, Quinzii CM, Kanki T, Rodenburg RJ, Naini A (2006). Leigh syndrome with nephropathy and CoQ10 deficiency due to decaprenyl diphosphate synthase subunit 2 (PDSS2) mutations. Am J Hum Genet.

[29] Quinzii C, Naini A, Salviati L, Trevisson E, Navas P, Dimauro S (2006). A mutation in para-hydroxybenzoate-polyprenyl transferase (COQ2) causes primary coenzyme Q10 deficiency. Am J Hum Genet.

[30] Diomedi-Camassei F, Di Giandomenico S, Santorelli FM, Caridi G, Piemonte F, Montini G (2007). COQ2 nephropathy: a newly described inherited mitochondriopathy with primary renal involvement. J Am Soc Nephrol.

[31] Mitsui J, Matsukawa T, Yasuda T, Ishiura H, Tsuji S (2016). Plasma coenzyme Q10 levels in patients with multiple system atrophy. JAMA Neurol.

